# Baseline PET/CT imaging parameters for prediction of treatment outcome in Hodgkin and diffuse large B cell lymphoma: a systematic review

**DOI:** 10.1007/s00259-021-05233-2

**Published:** 2021-02-18

**Authors:** R. Frood, C. Burton, C. Tsoumpas, A. F. Frangi, F. Gleeson, C. Patel, A. Scarsbrook

**Affiliations:** 1 Department of Nuclear Medicine, Leeds Teaching Hospitals NHS Trust, Leeds, UK; 2 Leeds Institute of Health Research, University of Leeds, Leeds, UK; 3 Department of Haematology, Leeds Teaching Hospitals NHS Trust, Leeds, UK; 4 Leeds Institute of Cardiovascular and Metabolic Medicine, University of Leeds, Leeds, UK; 5 Centre for Computational Imaging and Simulation Technologies in Biomedicine (CISTIB), School of Computing and School of Medicine, University of Leeds, Leeds, UK; 6 Medical Imaging Research Center (MIRC), University Hospital Gasthuisberg, KU Leuven, Leuven, Belgium; 7 Department of Radiology, Oxford University Hospitals NHS Foundation Trust, Oxford, UK

**Keywords:** Diffuse large B-cell lymphoma, Hodgkin lymphoma, PET-CT, Outcome prediction, Radiomics

## Abstract

**Purpose:**

To systematically review the literature evaluating clinical utility of imaging metrics derived from baseline fluorine-18 fluorodeoxyglucose positron emission tomography/computed tomography (PET/CT) for prediction of progression-free (PFS) and overall survival (OS) in patients with classical Hodgkin lymphoma (HL) and diffuse large B cell lymphoma (DLBCL).

**Methods:**

A search of MEDLINE/PubMed, Web of Science, Cochrane, Scopus and clinicaltrials.gov databases was undertaken for articles evaluating PET/CT imaging metrics as outcome predictors in HL and DLBCL. PRISMA guidelines were followed. Risk of bias was assessed using the Quality in Prognosis Studies (QUIPS) tool.

**Results:**

Forty-one articles were included (31 DLBCL, 10 HL). Significant predictive ability was reported in 5/20 DLBCL studies assessing SUVmax (PFS: HR 0.13–7.35, OS: HR 0.83–11.23), 17/19 assessing metabolic tumour volume (MTV) (PFS: HR 2.09–11.20, OS: HR 2.40–10.32) and 10/13 assessing total lesion glycolysis (TLG) (PFS: HR 1.078–11.21, OS: HR 2.40–4.82). Significant predictive ability was reported in 1/4 HL studies assessing SUVmax (HR not reported), 6/8 assessing MTV (PFS: HR 1.2–10.71, OS: HR 1.00–13.20) and 2/3 assessing TLG (HR not reported). There are 7/41 studies assessing the use of radiomics (4 DLBCL, 2 HL); 5/41 studies had internal validation and 2/41 included external validation. All studies had overall moderate or high risk of bias.

**Conclusion:**

Most studies are retrospective, underpowered, heterogenous in their methodology and lack external validation of described models. Further work in protocol harmonisation, automated segmentation techniques and optimum performance cut-off is required to develop robust methodologies amenable for clinical utility.

**Supplementary Information:**

The online version contains supplementary material available at 10.1007/s00259-021-05233-2.

## Background

Lymphoma is a haematopoietic malignancy, which can be broadly categorised into Hodgkin and non-Hodgkin disease. Hodgkin lymphoma (HL) accounts for approximately 10% of all newly diagnosed cases, and its hallmark is the presence of Hodgkin and Reed–Sternberg (HRS) cells [1]. HL can be further sub-divided based on morphology and immunohistochemistry into classical Hodgkin lymphoma (cHL), which has four further sub-categories, or nodular lymphocyte-predominant Hodgkin lymphoma (NLPHL) [1]. The majority (90%) of disease is due to cHL. HL is associated with a good prognosis having an overall 5-year survival of 86.6% [2]. Non-Hodgkin lymphoma (NHL) is the most prevalent form of lymphoma with over 50 sub-types, the most common being diffuse large B cell lymphoma (DLBCL) [3]. The overall 5-year survival rate is 72% for NHL but this varies by stage and subtype [2]. DLBCL has a 5-year survival of approximately 60–80%, which has improved since the use of anthracycline-containing chemotherapy and rituximab (R-CHOP) [2, 4].

There are several pretreatment clinical prognostic tools developed to stratify both DLBCL and HL. In 1993, Shipp et al. introduced the international prognostic index (IPI) for predicting overall survival in DLBCL patients based on a retrospective study of 2031 patients treated with CHOP. The IPI has been further refined with an age-adjusted version (aa-IPI), a revised version developed following the use of R-CHOP (R-IPI), and a version based on the National Comprehensive Cancer Network database (NCCN-IPI). HL disease can be split into early (stage I and II) or advanced (stage III or stage IV) with early being split into favourable or unfavourable depending on one of the many scoring systems including, but not limited to, the German Hodgkin Study Group (GHSG), European Organisation of Research and Treatment of Cancer (EORTC), Groupe d’Etudes des Lymphomes de l’Adulte (GELA), National Cancer Institute (NCI) or National Comprehensive Cancer Network 2010 (NCCN 2010) scores. However, given the variation in the prognostic groups derived from the different scoring systems, further information obtained from imaging may improve prognostication.

2-deoxy-2-[Fluorine-18]fluoro-D-glucose (FDG) positron emission tomography/computed tomography (PET/CT) is widely used for staging and response assessment in HL and NHL [5]. Response assessment PET/CT studies are performed at various time points, including during and after treatment [5]. The parameter most commonly used in assessment is the standardised uptake value (SUV) at sites of disease, which is compared to physiological activity in reference areas such as the mediastinal blood pool and liver and is reported using an ordinal (qualitative) scale (Deauville Score (DS)).

A variety of imaging-derived quantitative parameters have been reported in the literature with potential utility for predicting prognosis or treatment outcome. These metrics range from those based on tumour volume to metabolic features, including shape and texture. At present, none have been translated into routine clinical practice. The purpose of this study was to perform a systematic review of the literature reporting the use of quantitative imaging parameters derived from pretreatment FDG PET/CT for prediction of treatment outcome for HL and DLBCL. Due to the varied nature of NHL, DLBCL was chosen as it is the most common subtype of NHL.

## Methods

### Search strategy and selection criteria

A search of MEDLINE/PubMed, Web of Science, Cochrane, Scopus and clinicaltrials.gov databases was performed for articles on PET/CT imaging parameters in lymphoma treatment assessment. The search strategy included three primary operator criteria linked with the “AND” function. The first criteria consisted of “lymphoma”, the second of “PET” or “positron emission tomography”, and the third of “outcome”, “prognosis”, “prediction”, “parameter”, “radiomics”, “machine learning”, “deep learning” or “artificial intelligence”. Case studies, articles not published in English, phantom studies, studies not assessing treatment outcomes using baseline imaging in HL or DLCBL, studies assessing primary anatomical presentations of lymphoma or HIV-related lymphoma, mixed pathology studies and studies assessing novel treatments were excluded. After duplications were excluded, studies were screened for eligibility based on the title, abstract and subsequently on full text. The references of the articles included in the systematic review were manually reviewed to identify further publications which met the inclusion criteria. The results were stored in bibliographic management software. Preferred Reporting Items for Systematic Reviews and Meta-Analysis (PRISMA) criteria were adhered to [6].

### Quality assessment

The Quality in Prognosis Studies (QUIPS) tool was used to evaluate validity and bias which considers six areas: inclusion, attrition, prognostic factor measurement, confounders, outcome measurement, and analysis and reporting [7]. Prompting questions and modifications applied to the QUIPS tool are detailed in Supplemental Table 1. Two authors (RF and AS) independently reviewed all studies which met inclusion criteria and scored each of the six domains as high, moderate or low risk of bias. Any discrepancies were agreed in consensus. Overall risk of bias for each paper was further categorised based on the following criteria: if all domains were classified as low risk, or there was up to one moderate risk, the paper was classified as low risk of bias. If one or more domains were classified as high risk, the paper was classified as high risk of bias. All papers in between were classified as having moderate risk of bias [8].

## Results

**Fig. 1:**
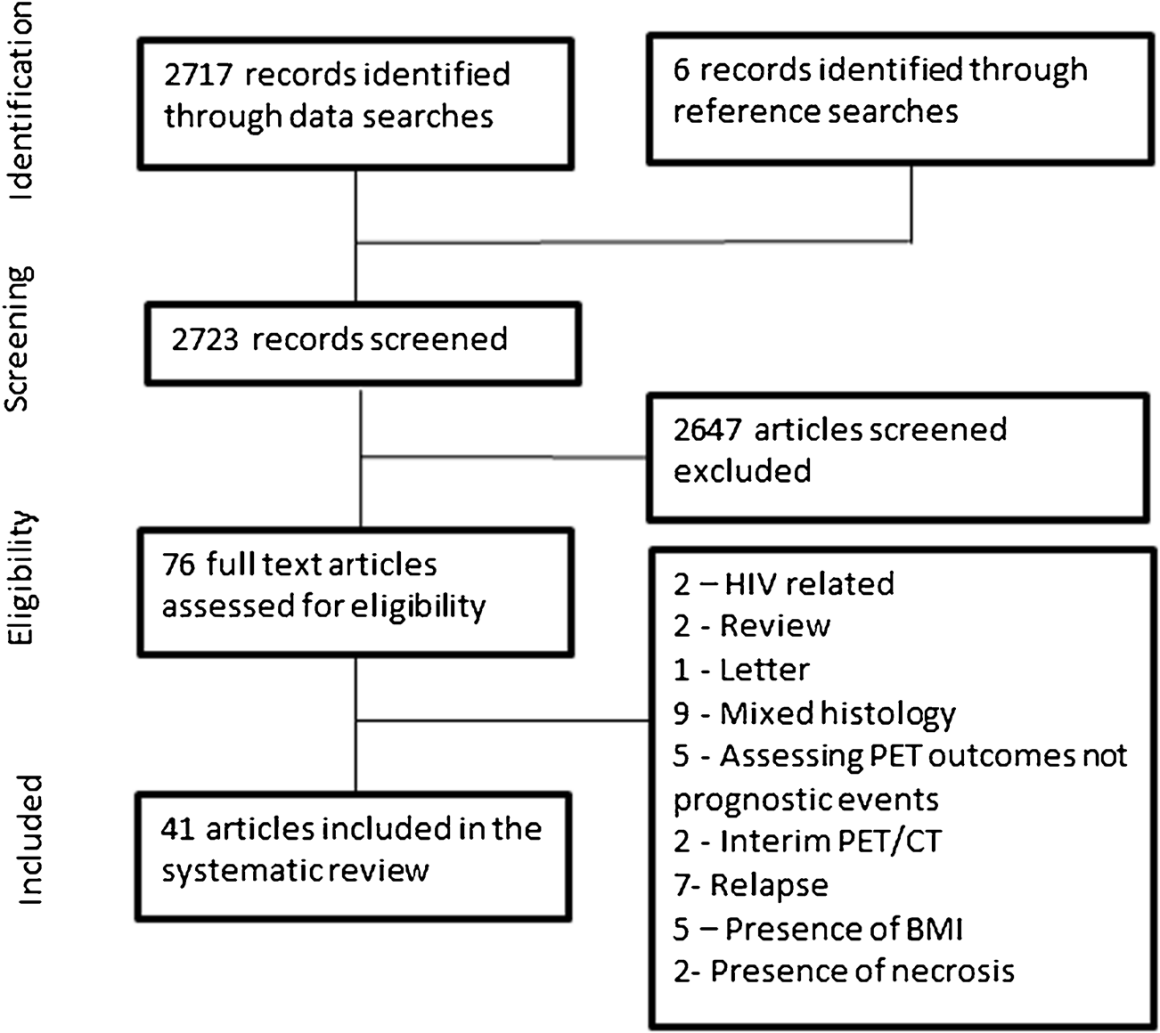
PRISMA flow diagram illustrating the methodology for study selection for the systematic review of lymphoma imaging parameters. *BMI* bone marrow involvement, *Relapse* indicates studies investigating previously treated cases

Results are current to July 2020. The database search strings yielded 2717 results after duplicates were excluded. Following screening and assessment of eligibility, 41 articles meeting the study inclusion criteria were included. Figure 1 details the study selection.

### Quality assessment

No studies showed low risk of bias in all six domains (Supplemental Table 2). Only two studies demonstrated a low risk for participation; no studies had a low risk in attrition, prognostic measurement, outcome measurement or confounding factors; 33 studies had low risk for analysis and reporting. All studies were assessed as having either moderate (24/41, 59%) or high (17/41, 41%) overall risk of bias. Of the high risk studies, 6 had high risk scores of bias in participation, 5 in attrition, 8 in prognostic measurement, 8 in outcome measurement, 10 in confounding factors and 7 in analysis and reporting categories.

All studies were retrospective, with 28/41 single centre. Six reports were based on retrospective analysis of trial data from prospective studies. Four studies stated that they were compliant with the European Association of Nuclear Medicine (EANM) guidelines with their scanning protocol; 10/41 did not take into consideration important co-founders such as different treatment regimes, stage, prognostic scores or histology. Only six studies defined the method for calculation of SUV, and 7 studies used a validation cohort to test the predictive models (Table 1). Of the radiomic studies, one study referenced the image biomarker standardisation initiative (IBSI) within the discussion but none of the papers explicitly stated that they had complied with IBSI guidelines.

**Table 1:** Overview of study design and risk of bias for each of the studies included in the systematic review

Study	Prospective	Multi-centre	PET scanners used	EANM guidelines stated	SUV defined	Definition of prognostic factor provide	Follow up period	Separate validation cohort	Overall risk of bias
Adams [9]	N	N	Siemens Biograph 40 TruePoint	N	N	PFS - relapse/progression/death attributable to PFS OS - Death from any cause	Median: 994 days	N	Moderate
Aide [10]	N	N	Siemens Biograph TrueV	N	N	EFS - relapse/progression/unplanned treatment/death attributable to PFS	2-year EFS	Y	High
Aide [11]	N	N	Siemens Biograph TrueV	Y	N	PFS - relapse/progression OS - death from lymphoma or treatment	Median: 25.7 months	N	Moderate
Akhtari [12]	N	N	GE Discovery ST GE Discovery RX GE Discovery STE	N	Y(bw)	FFP - relapse or refractory disease OS - death from any cause	Median: 4.96 years	N	Moderate
Albano [13]	N	Y	GE Discovery ST GE Discovery 690	Y	N	PFS - progression/relapse/death OS - death from any cause	Median: 40 months	N	Moderate
Angelopulou [14]	N	N	Multiple not defined	N	N	FFP - relapse or refractory disease OS - death from any cause	Median: 56 months	N	High
Capobianco [15]	N	Y	Multiple^1^	N	N	Not defined	Median: 5 years	Y	High
Ceriani [16]	N	Y	Multiple not defined	N	N	Not defined	Median: 64 months, 34 months	Y	High
Chang [17]	N	N	GE Discovery ST	N	N	PFS - progression/relapse/death OS - death from any cause	Median: 28.7 months	N	Moderate
Chang [18]	N	N	GE Discovery ST	N	N	PFS - progression/relapse/death OS - death from any cause	median 36 months	N	Moderate
Chihara [19]	N	N	GE Discovery LS	N	Y(bw)	PFS - progression/relapse/death from any cause OS - death from any cause	Median: 34.4 months	N	Moderate
Cottereau [20]	N	Y	Multiple not defined	N	N	Not defined	Median: 44 months	N	High
Cottereau [21]	N	Y	Multiple not defined	N	N	PFS - progression/death from any cause OS - death from any cause	Median: 55 months	Y	Moderate
Cottereau [22]	N	N	Siemens Biograph 16	N	N	OS and PFS were defined according to the revised NCI criteria	Median: 64 months	N	Moderate
Decazes [23]	N	N	Siemens Biograph Sensation 16 HiRes	N	N	Both OS and PFS were defined according to the revised NCI criteria	Median: 44 months	N	Moderate
Esfahani [24]	N	N	Siemens Biograph	N	N	PFS - recurrence	Mean: 51 months	N	High
Gallicchio [25]	N	N	GE Discovery VCT GE Discovery LS VCT	N	N	Progression/disease-related death	Median: 18 months	N	High
Huang [26]	N	N	GE Discovery LS	N	Y(bw)	PFS - progression/relapse/death OS - death from any cause	Median: 30 months	N	Moderate
Ilyas [27]	N	N	GE Discovery ST GE Discovery VCT	N	N	PFS - progression/death from any cause OS - death from any cause	Median: 3.8 years	N	High
Jegadesh [28]	N	N	Not defined	N	N	Not defined	Median: 43.9 months	N	Moderate
Kanoun [29]	N	N	Philips Gemini GXL Philips Gemini TOF	N	N	PFS - progression/relapse/death from any cause	Median: 50 months	N	High
Kim [30]	N	N	Siemens Biograph 6	N	N	EFS - relapse/progression/stopping of treatment/death from any cause OS - death from any cause	Median: 27.8 months	N	Moderate
Kim [31]	N	N	Philips Gemini Siemens Biograph 40	N	N	PFS - progression/relapse/death OS - death (? any cause)	Median: 25.8 months	N	Moderate
Kwon [32]	N	Y	GE Discovery ST	N	Y(bw)	PFS - progression/relapse/death from any cause OS - death from any cause	Median: 30.8 months	N	High
Lanic [33]	N	Y	Siemens Biograph LSO Sensation 16	N	N	PFS - progression/relapse/death from any cause OS - death from any cause	Median: 28 months	N	High
Lue [34]	N	N	GE Discovery ST	N	N	PFS - progression/relapse/death from any cause OS - death from any cause	Median: 48 months	N	Moderate
Mettler [35]	N	Y	Multiple not defined	N	N	PFS - progression/relapse/death from any cause OS - death from any cause	Not defined	N	High
Mikhaeel [36]	N	N	GE Discovery ST GE Discovery VCT	N	N	PFS - progression/death from any cause OS - death	Median: 3.8 years	N	Moderate
Milgrom [37]	N	N	GE Discovery ST GE Discovery RX GE Discovery STE	N	N	Relapse or progression or death	Not defined	Y	High
Miyazaki [38]	N	N	GE Discovery STE	N	N	PFS - relapse/death from any cause OS - death	Median: 32.7 months	N	Moderate
Park [39]	N	N	GE Discovery LS, GE Discovery STE	N	N	PFS - progression/relapse/death from any cause OS - death from any cause	Median: 21 months	N	High
Sasanelli [40]	N	Y	Philips Gemini GXL Siemens Biograph 2 GE Discovery ST	N	Y(bw)	PFS - relapse OS - death from any cause	Median: 39 months	N	Moderate
Senjo [41]	N	Y	Philips Gemini GXL GE Discovery ST	Y	Y(bw)	PFS - progression/relapse/death OS - death	Median: 33.1 months, 32.8 months	Y	High
Song [42]	N	Y	Siemens Biograph	N	N	PFS progression OS - death from any cause	Median: 40.8 months	Y	Moderate
Song [43]	N	Y	Siemens Biograph	N	N	Not defined	Median: 45.8 months	N	Moderate
Song [44]	N	Y	Siemens Biograph	N	N	PFS progression, death related to lymphoma OS - death from any cause	Median: 36 months	N	Moderate
Toledano [45]	N	N	Siemens Biograph Sensation 16 HiRes	N	N	OS and PFS were defined according to the revised NCI criteria	Median: 40 months	N	Moderate
Tseng [46]	N	N	GE Discovery LS	N	N	Not defined	Median: 50 months	N	High
Xie [47]	N	N	Siemens Biograph 64	Y	N	PFS - progression/relapse/death from any cause	Median: 17 months	N	High
Zhang [48]	N	N	Siemens Biograph 64	N	N	PFS - progression, death related to lymphoma	Median: 34 months	N	Moderate
Zhou [49]	N	N	GE Discovery ST Siemens Biograph 64	N	N	N - not defined	Median: 30 months	N	Moderate

*PFS* progressive free survival; *EFS* event free survival; *OS* overall survival; *FFP* free from progression; *bw* body weight; *1* Discovery 690, STE, ST, RX, 600, 710, LS, Biograph HiRez, Truepoint, mCT, LSO, BGO and Gemini TF and GXL; *EANM* European Association of Nuclear Medicine

As there were no studies deemed to be of low risk for overall bias, a decision was made to include the high risk studies in the systematic review, as removal of these would introduce its own inherent bias.

## Metabolic parameters

SUV is the commonest metric extracted from PET studies. This represents a ratio of radioactivity at a given image location compared to injected whole-body radioactivity [50]. There are several iterations of SUV, including the maximum or mean SUV within a contoured area (SUVmax and SUVmean), or SUVpeak which is the average SUV of a region of interest centred on the highest uptake region within the contoured area. SUV supports other metabolic parameters such as metabolic tumour volume (MTV), which is the volume of disease contoured at a specified SUV threshold, and total lesion glycolysis (TLG), which is the MTV multiplied by SUVmean. Published evidence regarding metabolic parameters used in the pretreatment assessment of lymphoma is summarised below.

### SUV metrics for prediction of outcome

- DLBCL

**Fig. 2:**
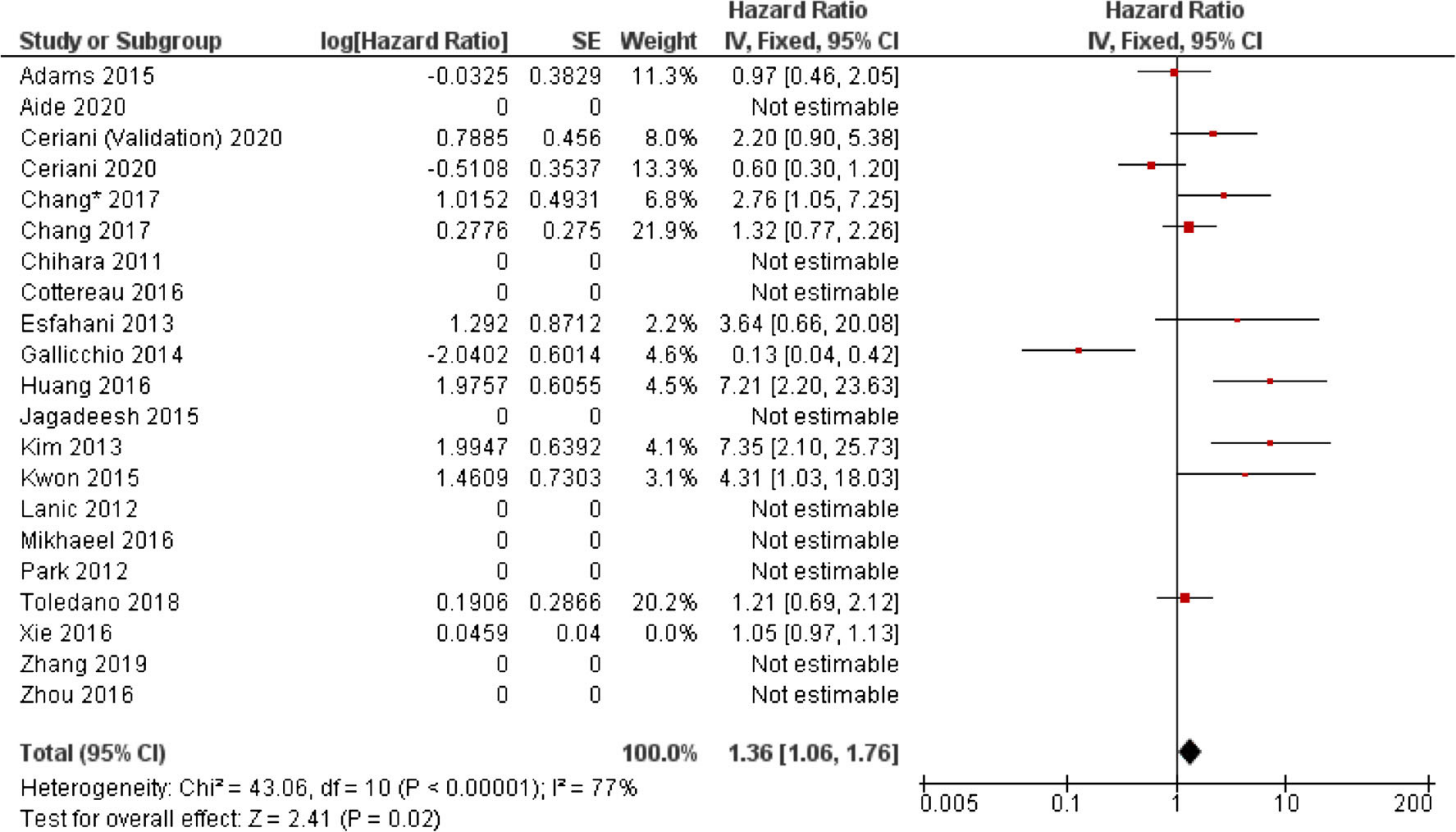
Forest plot demonstrating hazard ratios for progression-free/event-free survival for patients with DLBCL using a dichotomous cut-off value derived from SUVmax. Studies which do not provide hazard ratios are included but no estimate is given

**Fig. 3:**
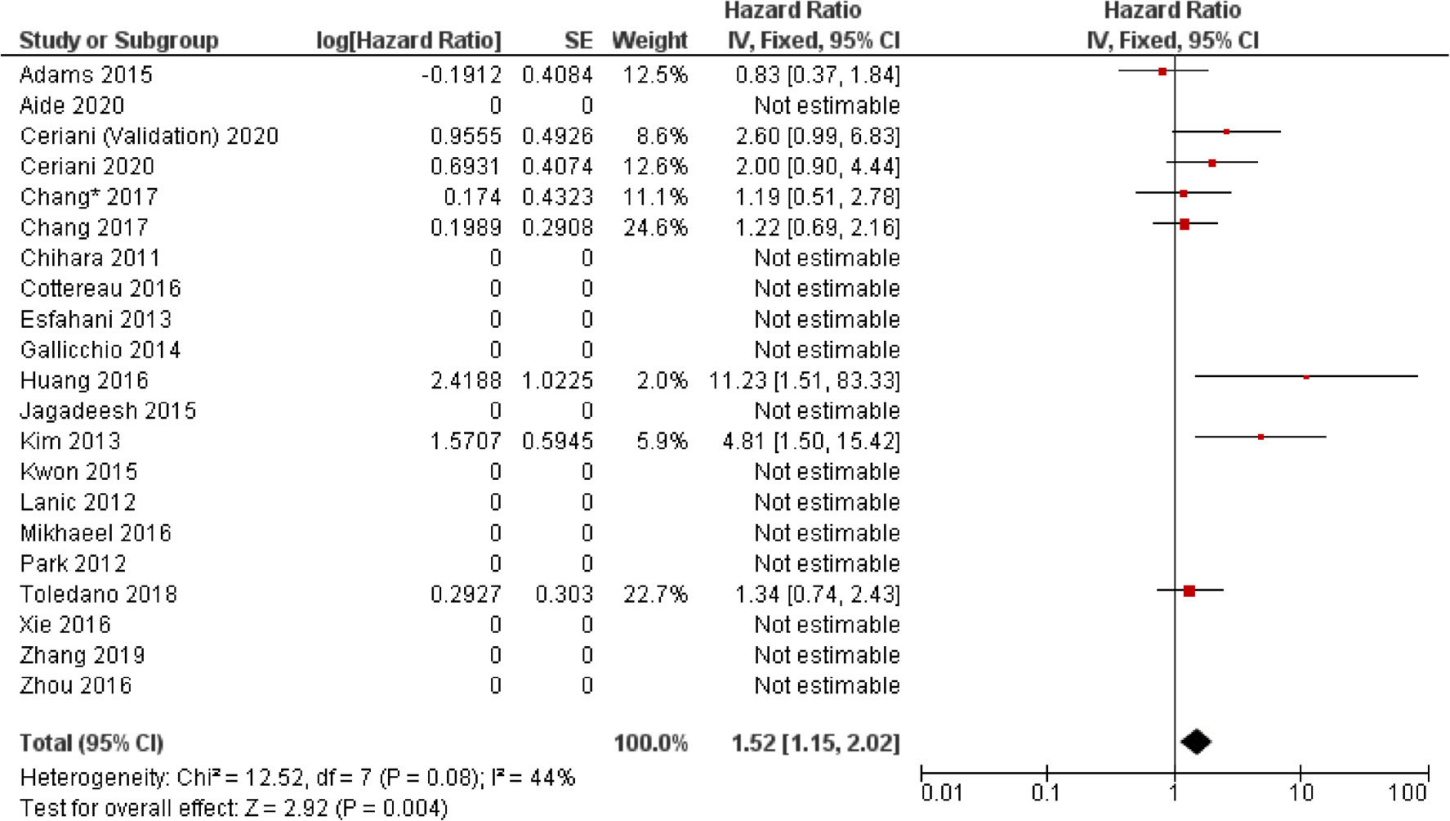
Forest plot demonstrating hazard ratios for overall survival for patients with DLBCL using a dichotomous cut-off value derived from the SUVmax. Studies which do not provide hazard ratios are included but no estimate is given

The majority of studies assessing the use of baseline SUVmax in DLBCL report no significant ability to predict progression-free survival (PFS) or overall survival (OS) (Table 2). Forest plots illustrating hazard ratios (HR) for PFS and OS are demonstrated in Figs. 2 and 3. From the results included in the forest, the overall HR was 1.35 (CI 95% 1.06–1.76) for PFS and 1.52 (CI 95% 1.15–2.02). However, there is considerable heterogeneity specifically in the PFS analysis (I^2^ = 77%) and reporting bias is present because a number of studies which did not report any significance did not provide the results required to calculate a HR.

**Table 2:** Studies assessing the use of standardised uptake value (SUV) in predicting outcomes in diffuse large B cell lymphoma (DLBCL) and Hodgkin lymphoma (HL)

First author	Year	Type	Study type	Patient no.	Stage		Treatment	Events (follow-up cut-off)	SUV Type	Cut-off value	Predictive univariate analysis HR (95% CI)		Predictive multivariate analysis HR (95% CI), parameters included in multivariate analysis	
					I/II	III/IV					PFS	OS	PFS	OS
Aide [10]	2020	DLBCL	R	132 (80:20 training/validation)	NR	NR	R-CHOP, R-ACVBP	Relapse/death: 102 (2-year)	SUVmax	32.21	NS	NR	NR	NR
Albano [13]^a^	2020	HL (Aged 65–92)	R	123	36	87	ABVD, BEACOPP, R-CHOP, ± RT, RT	Relapse: 51 Died: 37 (no defined cut-off)	L-L SUV R	9.3	0.447 (0.237–0.748)	0.526 (0.261–0.992)	0.228 (0.049–0.765)	0.200 (0.033–0.353)
									L-BP SUV R	6.4	0.469 (0.229–0.774)	0.523 (0.241–0.983)	0.354 (0.069–0.989)	0.555 (0.201–1.002)
Ceriani [16]	2020	DLBCL	R	141 – Testing	61	80	R-CHOP ± RT	NR	Max	20	NS	NS	NR	NR
				113 -Validation	49	64	R-CHOP ± RT	NR	Max	31	NS	NS	NR	NR
Zhang [48]	2019	DLBCL	R	85	32	53	R-CHOP/R-CHOP like	Relapse/Died:23 (3-year)	Max	NR – AUC 0.573	NR	NR	NR	NR
Akhtari [12]	2018	HL	R	267	205	62	ABVD ± RT/other^a^	Relapsed/refractory: 27 (5-year)	Max	NR	NS	NR	NR	NR
Cottereau [21]	2018	HL	R	258	258	0	ABVD ± RT	PFS: 27 events OS: 12 (5-year)	Max	NR	NS	NS	NR	NR
Toledano [45]	2018	DLBCL	R	114	26	88	R-CHOP/R-CHOP like	Relapse: 52 Died: 43 (5-year)	Max	NR	NS	NS	NR	NR
Angelopoulou [14]	2017	HL	R	162	76	86	ABVD ± BEACOPP, ± RT	PFS: 81% OS: 93% (5-year)	Max	<9, 9–18, >18	93%, 81%, 58%	NR	NR	NR
Chang [17]	2017	DLBCL	R	118	48	70	R-CHOP	Relapse: 55 Died: 49 (5-year)	Max	18.8	NS	NS	NR	NR
Chang [18]	2017	DLBCL	R	70	35	35	R-CHOP	NR	Tumour Max	19	2.76 (1.05–7.61)	NS	3.27 (1.11–9.60)	NS
									Sternal Max	1.6	NS	2.34 (1.01–5.44)	NS	2.62 (1.10–6.28
Cottereau [22]	2016	DLBCL	R	81	16	65	R-CHOP, R-ACVBP	Relapse: 34 (5-year)	Max	NR	NS	NS	NR	NR
Huang [26]	2016	DLBCL	R	140	62	78	R-CHOP/CHOP	PFS: 73.8% OS: 86.1% (30-month)	Max	9	7.2 (2.201–23.631)	11.4 (1.514–86.350 0.018)	4.7 (1.429–16.022 0.011)	NS
Mikhaeel [36]	2016	DLBCL	R	147	46	101	R-CHOP	PFS: 65.4% OS: 73.7% (5-year)	Max	Split into tertiles	NS	NS	NR	NR
Xie [47]	2016	DLBCL	R	60	12	48	R-CHOP	Relapse: 17 Died: 3 (40-month)	Max	NR	NS	NR	NS	NR
Zhou [49]	2016	DLBCL	R	91	34	57	R-CHOP	Relapse: 37 Died: 11 (5-year)	Max	PFS – 19 OS – 15.8	NS	NS	NR	NR
Adams [9]	2015	DLBCL	R	73	11	62	R-CHOP	Relapse: 27 Death: 24 (No defined cut-off)	Max	NR	NS	NS	NR	NR
Jagadeesh [28]	2015	DLBCL	R	89	0	89	R-CHOP/R + other	LR: 50% (5-year)	Max	15	NS for LR	NR	NS for LR	NR
Kwon [32]	2015	DLBCL	R	92	54	38	R-CHOP	Relapse: 33 Died: 3 (No defined cut-off)	Max	10.5	4.31 (1.03–18.1)	NR	NS	NR
Gallicchio [25]	2014	DLBCL	R	52	26	26	R-CHOP, R-COMP	Relapse: 15 Died: 2 (18 month)	Max	13.5	0.13 (0.04–0.46)	NR	NR	NR
Esfahani [24]	2013	DLBCL	R	20	8	12	R-CHOP	Relapse: 6 (No defined cut-off)	Max	13.84	NS	NR	NR	NR
									Mean	6.44	NS	NR	NR	NR
Kim [31]	2013	DLBCL	R	140	77	63	R-CHOP	Relapse: 21 Died: 16 (2-year)	Max	16.4	NS	NS	NR	NR
Lanic [33]	2012	DLBCL	R	57	NR	NR	R-CHOP, intensified R-CHOP	NR (2-year)	Max	NR	NS	NS	NR	NR
Park [39]	2012	DLBCL	R	100	55	45	R-CHOP	NR	Max	NR	NS	NS	NR	NR
									Sum	NR	1.011 (1.002–1.020)	1.016 (1.006–1.026)	NR	NR
Tseng [46]	2012	HL	R	30	11	19	Standford V, ABVD, VAMP, BEACOPP	Relapse =6 (4-year)	Max	NR	NS	NS	NR	NR
									Mean	NR	NS	NS	NR	NR
Chihara [19]	2011	DLBCL	R	110	65	45	R-CHOP ± RT	PFS: 75% OS: 84% (3-year)	Max	30	Sig.	Sig.	HR6.74	NS

*R* retrospective; *NR* not reported; *NS* not significant; *Sig.* significant; *HR* hazard ratio; *CI* confidence interval; *PFS* progressive free survival; *OS* overall survival; *R-CHOP* rituximab cyclophosphamide, doxorubicin hydrochloride, vincristine (Oncovin) and prednisolone; *R-ACVBP* Rituximab, Doxorubicin, Cyclophosphamide, Vindesine, Bleomycin, prednisolone; *R-COMP* prednisolone, Cyclophosphamide, Vincristine, Myocet and Rituximab; *RT* radiotherapy; *ABVD* doxorubicin (Adriamycin), bleomycin, vinblastine and dacarbazine; *eBEACOPP* escalated dose bleomycin, etoposide, doxorubicin (Adriamycin), cyclophosphamide, vincristine (Oncovin), procarbazine and prednisone; *VAMP* vincristine, doxorubicin hydrochloride, methotrexate, prednisolone

^a^The HRs presented as presented in the study but are inverse to the other HRs within the table

Of the studies which showed a prognostic ability for SUVmax, Gallicchio et al. reported this was the only imaging parameter able to predict PFS when compared to TLG and MTV in a small study of 52 DLBCL patients (26 early and 26 advanced stage) with a higher SUVmax associated with a longer PFS, the hazard ratio (HR) was 0.13 (0.04–0.46) [25]. A study by Kwon et al. assessing 92 DLBCL (54 stage I/II, 38 stage II/IV) patients reported that a SUVmax of 10.5 was significant in predicting PFS, but this was not an independent prognostic predictor at multivariate analysis with clinical factors such as age, Lactate Dehydrogenase (LDH) level, stage, IPI score or Eastern Cooperative Oncology Group (ECOG) status [32]. Conversely, Miyazaki et al. demonstrated that SUVmax was an independent predictor of 3-year PFS and R-IPI [38]. Chang et al. found that tumour SUVmax >19 was a significant predictor of 3-year PFS, whereas the SUVmax of sternal uptake was an independent predictor of 3-year OS in a study of 70 DLBCL patients [18]. The most extensive study evaluating SUVmax as a predictor of PFS and OS was performed by Ceriani et al. with a test cohort of 141 patients and a validation cohort of 113 patients, both containing a similar mix of stage and prognostic scores. SUVmax was not significant in predicting PFS or OS in either cohort [16].

b) HL

Five studies have assessed the use of SUVmax as a predictive parameter in HL patients with only one reporting significance (Table 2). The largest by Akharti et al. showed no significant ability of SUVmax to predict PFS and OS in 267 stage I and II HL patients (74 early favourable) [12]. These findings were concordant with a study by Cottereau et al.*,* who also found no significant ability of SUVmax to predict PFS or OS in 258 stage I and II patients. Angelopoulou et al. reported that SUVmax was a significant predictor of 5-year PFS in a study of 162 patients with a split of stages (stage I/II = 76, stage III/IV = 86) [14]. The cohort was stratified into three risk groups, SUVmax <9, 9–18 and > 18 with five-year PFS rate being 93%, 81% and 58% respectively, multivariate analysis was not performed. Albano et al. studied the prognostic ability of liver to lesion SUV ratio and blood pool to lesion ratio in 123 older (age > 65 years) HL patients [13]. They found that both parameters were significant (at univariate analysis) for PFS and OS. They also demonstrated these metrics to be independent prognostic markers when analysed with tumour stage, German Hodgkin Study Group (GHSG) risk group, MTV and TLG for PFS, and tumour stage, GHSG risk group and Deauville score for OS.

Factors affecting SUV such as scanner spatial resolution, image acquisition and PET reconstruction parameters combined with a relatively small number of events, variation in the number of early and advanced patients, differences in treatment and definition of PFS all influence the results [51, 52]. This is reflected by the variation in cut-off/threshold values used to risk-stratify patients within each of the studies.

### Metabolic tumour volume and total lesion glycolysis for prediction of outcome

- DLBCL

**Fig. 4:**
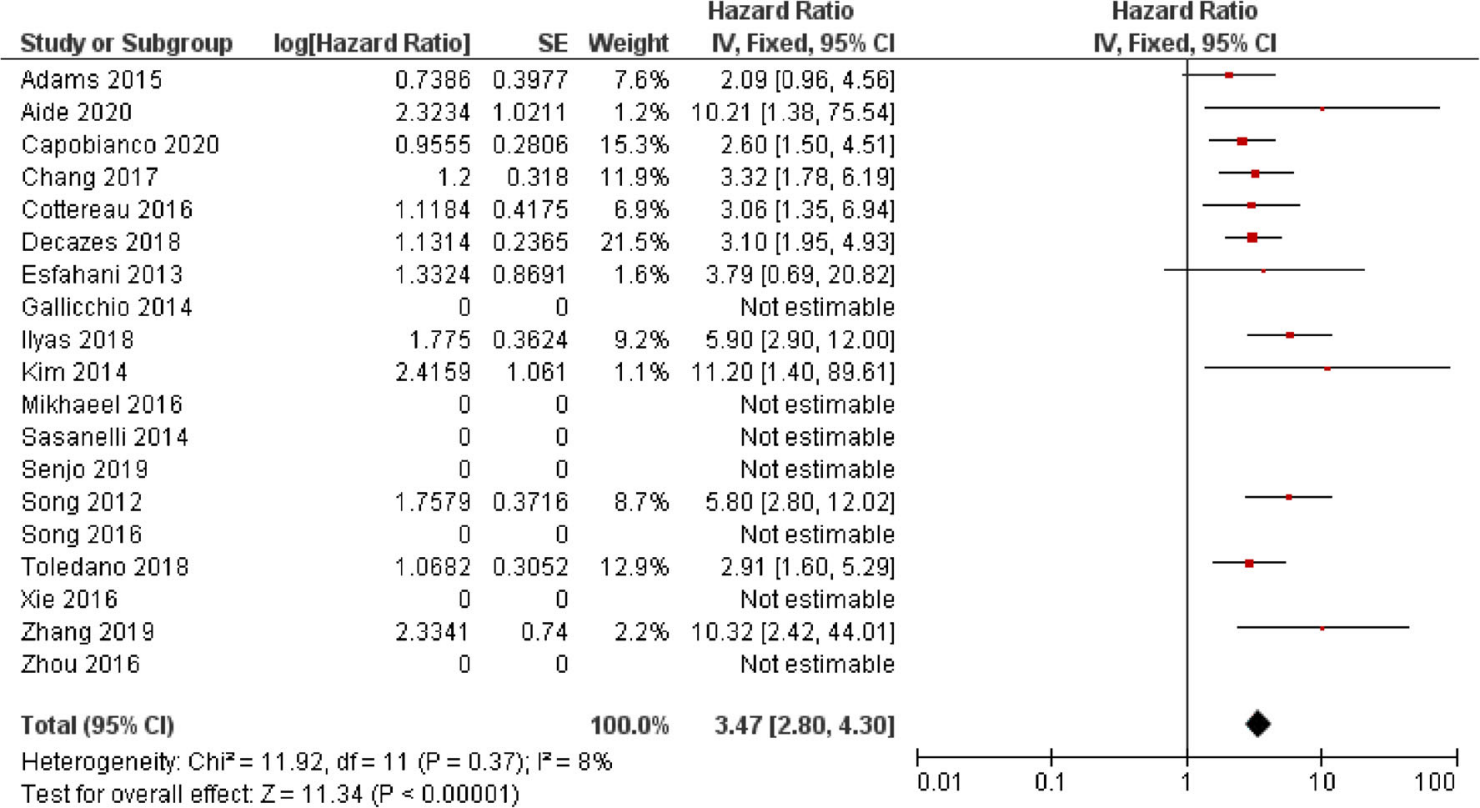
Forest plot demonstrating hazard ratios for progression-free survival for patients with DLBCL using a dichotomous cut-off value derived from the metabolic tumour volume. Studies which do not provide hazard ratios are included but no estimate is given

**Fig. 5:**
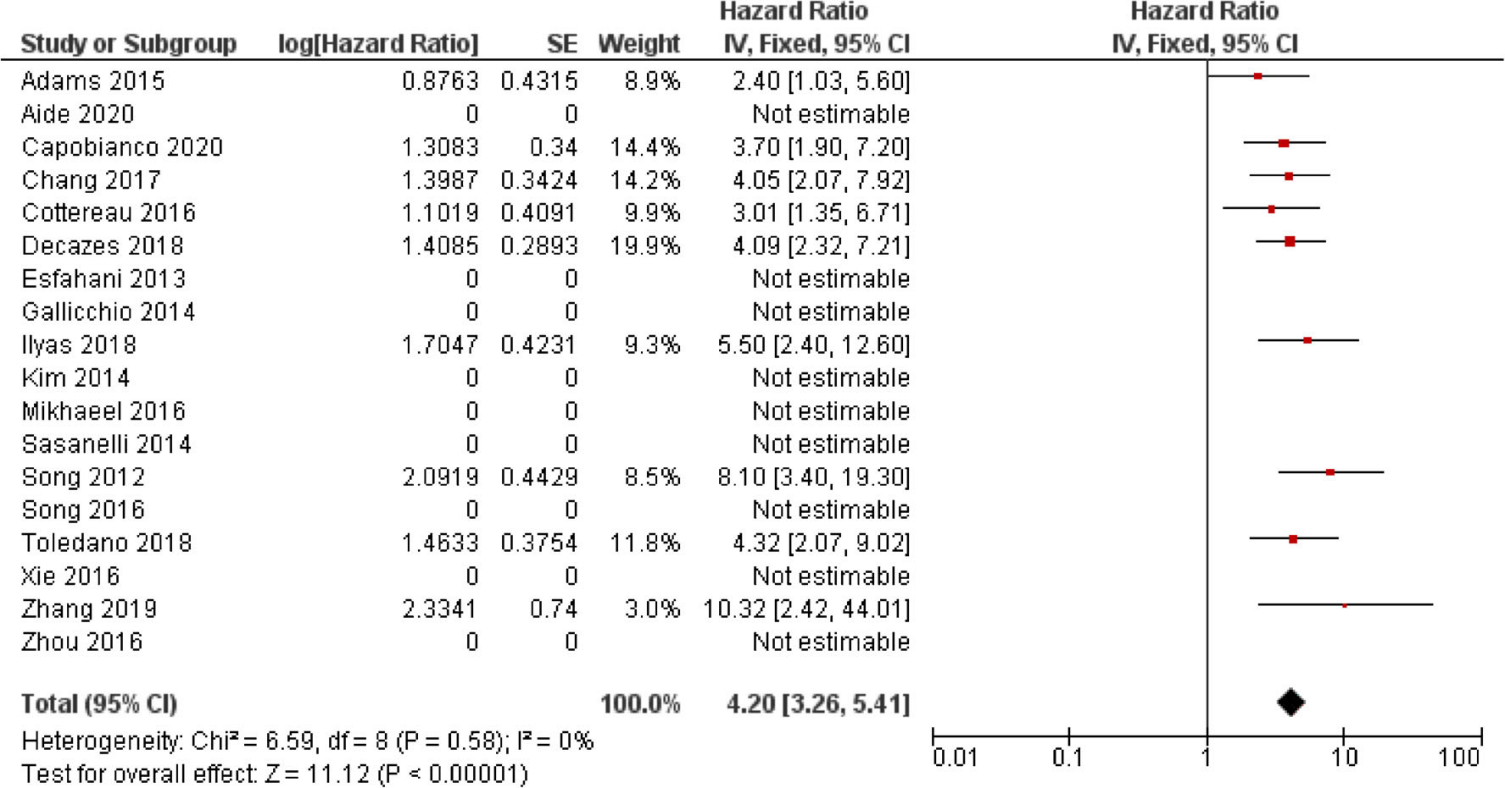
Forest plot demonstrating hazard ratios for overall survival for patients with DLBCL using a dichotomous cut-off value derived from the metabolic tumour volume. Studies which do not provide hazard ratios are included but no estimate is given

The potential utility of baseline MTV and TLG for predicting PFS and OS in patients with DLCBL has been reported in multiple studies (Table 3, Figs. 4 and 5). However, similar to SUVmax, there is heterogeneity in the cut-off values used which has led to variability in the reported survival rates between groups. Overall, the HR for MTV in PFS was 3.47 (CI 95% 2.80–4.30) and 4.20 (CI 95% 2.80–4.30) for OS. Again, reporting bias is present because a number of studies which did not report any significance did not provide the results required to calculate a HR.

**Table 3:** Studies assessing the use of metabolic tumour volume (MTV) and total lesion glycolysis (TLG) in predicting outcomes in diffuse large B cell lymphoma (DLBCL)

Author	Year	Patient no.	Stage		Treatment	Events (follow-up cut-off)	Segmentation threshold	MTV/ TLG	Suggested cut-off	Predictive univariate analysis HR (95% CI)		Predictive multivariate analysis HR (95% CI), parameters included in multivariate analysis	
			I/II	III/IV						PFS	OS	PFS	OS
Aide [10]	2020	132	NR	NR	R-CHOP, R-ACVBP	Relapse/death: 102 (2-year)	SUVmax of liver	MTV	111 ml	10.2 (1.4–75.5) (training data set) NS (Validation dataset)	NR	NS aa-IPI LZHGE	NR
Capobianco [15]	2020	280	26	264	R-CHOP	Relapse: 86 Died: 51 (4-year)	41% SUVmax	MTV	242 ml	NR	3.7 (1.9–7.2)	NR	NR
							CNN segmentation	MTV	110 ml	NR	2.8 (1.6–5.1)	NR	NR
Decazes [23]	2018	215	51	164	R-CHOP, R-CHOP like, R-ACVBP	Relapse: 92 Died: 74 (5-year)	41% SUVmax	MTV	487 ml	3.10(1.95–4.95)	4.09(2.32–7.21)	2.20 (1.26–3.83) IPI, chemotherapy, TVSR	2.78(1.41–5.48) IPI, chemotherapy, TVSR
Ilyas [27]	2018	147	46	101	R-CHOP	PFS: 65.4% OS: 73.7% (5-year)	PETTRA 2.5	MTV	PFS:396.1 ml OS: 457.8 ml	5.9 (2.9–12.2)	5.5 (2.4–12.5)	NR	NR
							HERMES 2.5	MTV	PFS:401.4 ml OS: 401.4 ml	5.9 (2.9–12.2 CI)	5.5 (2.4– 12.5)	NR	NR
							HERMES PERCIST	MTV	PFS:327.4 ml OS: 669.8 ml	4.8 (2.4–9.5 CI)	3.7 (1.8–7.8)	NR	NR
							HERMES 41%	MTV	PFS:165.7 ml OS: 189.3 ml	4.2 (2.2–7.9 CI) for	3.5 (1.8–7.0)	NR	NR
Senjo [41]	2019	150 (combined training and validation)	66	84	R-CHOP, R-THP-COP, R-CVP	Relapse 21 Died 48 (5-year)	>4.0 SUV	MTV	150 ml	NR	NR	2.49 (1.57–3.94)	2.75 (1.72–4.38)
Zhang [48]	2019	85	32	53	R-CHOP/R-CHOP-like	Relapse: 23 Died: 6 (3-years)		MTV	80.74 ml	10.32 (2.42–44.08)	NR	NR Correlated with TLG	NR
								TLG	1036.61 g	10.39 (2.43–44.39)	NR	10.42, (2.35–46.30)	NR
Toledano [45]	2018	114	26	88	R-CHOP/R-CHOP like	Relapse: 52 Died: 43 (5-year)	41% SUVmax	MTV	261.4 ml	2.91 (1.60–5.29)	4.32 (2.07–8.99)	2.05 (HR 1.02–4.15) GEP, IPI	2.70 (1.16–6.33) GEP, IPI
								TLG	1325.8 g	NS MC	4.82 (2.67–8.71)	NR	NR
Chang [17]	2017	118	48	70	R-CHOP	Relapse: 55 Died: 49 (5-year)	≥2.5 SUV	MTV	165.4 ml	3.32 (1.78–6.20)	4.05 (2.07–7.95)	2.31 (1.16–4.60) IPI	2.38 (1.12–5.04) Age, IPI
								TLG	1204.9 ml	2.57 (1.43–4.61)	2.96 (1.61–5.45)	NR	NR
Cottereau [22]	2016	81	16	65	R-CHOP, R-ACVBP	Relapse: 34 (5-year)	41% SUVmax	MTV	300 ml	3.06 (1.43–6.54)	3.01 (1.35–6.70)	1.61 (0.70–3.69)	3.0 (1.35–6.70)
								TLG	3904 g	2.92 (1.45–5.90)	2.39 (1.16–4.92)	NS	NS
Song [42]	2016	107*		107	R-CHOP	NR	≥2.5 SUV	MTV	601.2 ml	Sig.	Sig.	5.21 (2.54–10.69) IPI, bulky disease, BMI, IM MTV, CAs	5.33 (2.60–10.90) IPI, bulky disease, BMI, IM MTV, CAs
								IM MTV	260.5 ml	Significant	Significant	NS	NS
Zhou [49]	2016	91	34	57	R-CHOP	Relapse: 37 Died: 11 (5-year)	SUVmean of liver +3 SD	MTV	PFS: 70 ml OS: 78 ml	88 vs. 37%	98 vs. 60%	NS	NS
								TLG	PFS: 826.5 g OS: 726 g	83 vs. 34%	92 vs. 67%	5.21 (2.21–12.28) MTV, NCCN-IPI, Stage, B symptoms, LDH level Ki-67	9.1 (1.83–45.64) MTV, NCCN-IPI, Stage, B symptoms, LDH level Ki-67
Mikhaeel [36]	2016	147	46	101	R-CHOP	PFS5: 65.4% OS5: 73.7% (5-year)	41% SUVmax	MTV	Terties	Upper: 5.81 (2.38–14.14) Middle: 3.77 (1.49–9.51)	Sig.	Upper: 3.46 (1.10–10.86) Middle: 2.73 (0.89–8.40)	NR
								TLG	Tertiles	Upper: 4.90 (2.11- 11.38) Middle: (2.96 1.24 7.10)	Sig.	NR	NR
Xie [47]	2016	60	12	48	R-CHOP	Relapse: 17 Died: 3 (40 months)	SUVmean of liver +2SD	MTV	Continuous	1.030 (1.017–1.044)	NR	1.028 (1.014–1.043) NCCN-IPI	NR
								TLG	Continuous	1.078 (1.042–1.116)	NR	1.071 (1.032–1.112) NCCN-IPI	NR
Adams [9]	2015	73	11	62	R-CHOP	Relapse: 27 Death: 24 (No defined cut-off)	40% SUVmax	MTV	445 ml	NS	2.40 (1.03–5.60)	NR	NS NCCN-IPI
								TLG	4897.5 g	NS	NS	NR	NR
Kim [30]	2014	96	49	47	R-CHOP	PFS3: 69.5% OS3: 72.9% (No defined cut-off)	≥2.5 SUV	MTV	130.7 ml	11.2 (1.4–88.1)	NR	10.4 (1.3–83.4) IPI >/equal to 3 NS with IPI as individual parameters	NR
Gallicchio [25]	2014	52	41	11	R-CHOP like	Relapse: 15 Death: 2 (18 months)	42% SUVmax	MTV	16.1 ml	NS	NR	NR	NR
								TLG	589.5 g	NS	NR	NR	NR
Sasanelli [40]	2014	114	20	94	R-CHOP/R-ACVBP	Relapse: 31 Died: 25 (3-year)	41% SUVmax	MTV	550 ml	77 vs. 60%	87% vs. 60%/59% vs. 78% vs. 84% vs. 93%	NS	4.70 (1.82–12.18) Stage, LDH, Bulky disease 4.11 (1.67–10.16) aa-IPI, bulky disease
								TLG	4576 g	NS	64 vs. 85%	NR	NR
Esfahani [24]	2013	20	8	12	R-CHOP	Relapse: 6 Died: 0 (No defined cut-off)	50% SUVmax	MTV	379.2 ml	NS	N/A	NR	n/A
								TLG	704.8 g	11.21 (1.29–97)	N/A	NR	N/A
Kim [31]	2013	140	77	63	R-CHOP	Relapse: 21 Died: 16 (2-year)	25, 50 and 75% SUVmax	TLG25	817.8 g	2.8 (1.1–7.1)	NS	NR	NR
								TLG50	415.5 g	3.6 (1.3–10.0)	3.3 (1.0–10.0)	3.6 (1.3–10.0) IPI (2 splits)	3.1 (1.0–9.6) IPI(2 splits)
								TLG75	102.0 g	3.5 (1.3–9.5)	NS	NR	NR
Park [39]	2012	100	55	45	R-CHOP	NR	Blood Pool threshold	TLG	NR	NS	NR	NR	NR
Song [44]	2012	169	100	69	R-CHOP	PFS: 73.4% OS: 76.3 (3-year)	≥2.5 SUV	MTV	220 ml	5.80 (2.79–12.06)	8.10 (3.40–19.31)	5.30 (2.51–11.16) Stage 3	7.01 (2.90–16.93) Stage 3

*NR* not reported; *NS* not significant; *Sig.* significant; *HR* hazard ratio; *CI* confidence interval; *PFS* progressive free survival; *OS* overall survival; *R-CHOP* rituximab cyclophosphamide, doxorubicin hydrochloride, vincristine (Oncovin) and prednisolone; *R-ACVBP* Rituximab, Doxorubicin, Cyclophosphamide, Vindesine, Bleomycin, prednisolone; *R-THP-COP* rituximab, pirarubicin, cyclophosphamide, vincristine and prednisolone; *R-CVP* rituximab, cyclophosphamide, vincristine, prednisolone; *BMI* bone marrow involvement; *aa-IPI* age-adjusted International Prognostic Index; *NCCN-IPI* National Comprehensive Cancer Network – International Prognostic Index; *IM* intramedullary; *CAs* cytogenetic abnormalities; *LZHGE* Long-Zone High Grey-level Emphasis

One of the largest studies by Song et al. evaluated 169 patients with DLBCL (stage II and III without extranodal disease) treated with R-CHOP [44]. Patients with an MTV of <220cm^3^ had significantly better PFS and OS; 89.8 versus 55.6%, and 93.2 versus 58.0%, respectively [44]. MTV was predictive of PFS and OS regardless of stage. MTV remained significant when assessed using multivariate Cox regression with stage III disease, HR = 5.30 (95% 2.51–11.16) and HR = 7.01 (2.90–16.93) for 3-year PFS and 3-year OS, respectively. In another study, Song et al. reported that MTV was a prognostic predictor in 107 patients with bone marrow involvement (BMI); patients with an MTV of >601.2cm^3^ and BMI had worse PFS and OS survival compared to those with a smaller MTV and BMI [42]. Again, this was demonstrated to be an independent predictor when analysed with IPI, bulky disease, BMI, involved marrow MTV and > 2 cytogenetic abnormalities with an HR = 5.21 (95% CI 2.54–10.69) and HR = 5.33 (95% CI 2.60–10.90) for PFS and OS, respectively. However, there was no significant difference in survival between the smaller MTV with BMI group and a comparison cohort of patients without BMI. MTV summarises disease burden; however, it does not account for spread. Cottereau et al. studied four different spatial metrics besides TLG and MTV in 95 DLBCL patients on baseline scans to determine if a predictive model could be created [20]. The spatial parameters consisted of Dmax (distance between two of the furthest lesions), Dmax bulk (distance between the largest lesion and furthest lesion away from this), SPREADbulk (sum of all distances between bulky lesions) and SPREAD (sum of all distances between lesions). They found that a model combining MTV and Dmax could significantly distinguish between three prognostic groups. The low-risk group with an MTV <394cm^3^ and a Dmax <58 cm had a 4-year PFS of 94% and OS of 97%, the intermediate group with either an MTV >394cm^3^ or a Dmax >58 cm had a 4-year PFS of 73% and OS of 88% and the high-risk group with a MTV >394cm^3^ and a Dmax >58 cm had a 4-year PFS of 50% and OS of 53%.

Zhou et al. reported that although high baseline MTV and TLG were associated with poorer prognosis, only TLG was an independent predictor of PFS and OS in a study of 91 patients [49]. In this study, patients who demonstrated complete or partial remission were more likely to relapse if they had a high baseline TLG (40 versus 9%, *p* = 0.012). A possible explanation for the discrepancy between the prognostic ability of MTV and TLG in this study may be related to the correlation between MTV and TLG, confounded by relatively small sample sizes. Kim et al. evaluated TLG calculated using different MTVs derived using 25, 50 and 75% SUVmax thresholds in a mixed cohort (*n* = 140) of early and advanced stage DLBCL patients being treated with R-CHOP [31]. They found that all methods for calculating TLG were predictive of 2-year PFS, but only TLG50 was predictive of 2-year OS. Ilyas et al. also studied variation in segmentation technique and its potential to impact on predicting outcome in 147 DLBCL patients (46 stage I/II, 101 stage III/IV) all treated with R-CHOP [27]. The four segmentation techniques consisted of a threshold of SUV 2.5 on two software packages (PETTRA and Hermes), 41% SUVmax on Hermes software and an uptake higher than SUVmean of a 3-cm^3^ region of interest (ROI) within the right lobe of the liver (PERCIST) using the Hermes software. They found a strong agreement between all four methods, with the lowest intraclass coefficient being between PERCIST and 41% SUVmax thresholds being 0.86. They also reported similar receiver operator curves (ROC) between the four methods with the area under the curve (AUC) ranging from 0.74 to 0.76 for PFS, and 0.71 to 0.75 for OS. All four methods were significant predictors of PFS and OS. However, as stated in the paper, no method is likely to apply to all patients generally. Large heterogeneous masses are likely to be undersized with percentage thresholds, low uptake lesions may be missed using a standard threshold method and disease involving the liver may impede its use as the background value. This may have a more significant impact when further metrics are introduced, such as those based on texture when the size of the contour can also influence the reported values. The segmentation technique of choice also needs to be easily replicated. Recently, Capobianco et al. assessed the use of artificial intelligence (AI) using a convolutional neural network (CNN) to segment the MTV [15]. They found that AI-derived MTV correlated with reference MTV derived by two independent readers with a classification accuracy of 85%. Automatic segmentation is a key step required to enable implementation of MTV or TLG into clinical practice.

### HL

**Fig. 6:**
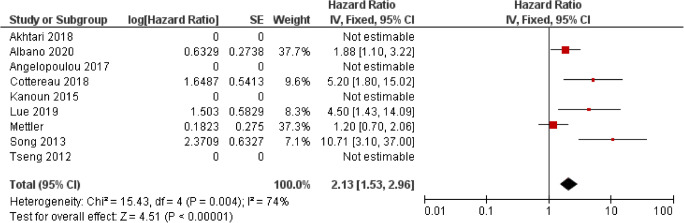
Forest plot demonstrating hazard ratios for progression-free survival for patients with HL using a dichotomous cut-off value derived from the metabolic tumour volume. Studies which do not provide hazard ratios are included but no estimate is given

**Fig. 7:**
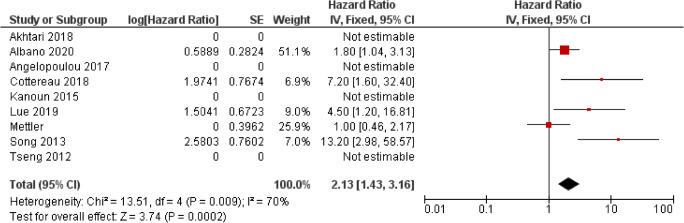
Forest plot demonstrating hazard ratios for overall survival for patients with HL using a dichotomous cut-off value derived from the metabolic tumour volume. Studies which do not provide hazard ratios are included but no estimate is given

Fewer studies have investigated the predictive ability of MTV and TLG in HL patients than in DLBCL (Table 4, Figs. 6 and 7). This is likely due to the higher survival rate of HL limiting the number of events demonstrated in a single centre and the variation in treatments and scoring systems for a favourable and unfavourable disease, which affect multi-centre studies. The majority of studies involved patients on an adaptive ABVD treatment regime, and results may not be transferrable to patients being treated with an adaptive BEACOPP regime. This confounding issue was highlighted in a study by Mettler et al. who assessed the prognostic ability of MTV in 310 patients with advanced HL being treated with eBEACOPP using four different contouring methods involving summation of the volume of each disease site using different defined thresholds: 41% SUVmax of each disease site, a threshold of liver SUVmax, a threshold of liver SUVmean and a fixed threshold of 2.5 SUV [35]. They found that MTV was predictive of interim PET response regardless of segmentation methodology; however, none was able to predict OS and PFS reliably. The divergent findings compared to previous studies are likely related to low event numbers and using a different treatment regime. Albano et al. demonstrated the significant ability of both MTV and TLG derived from 41% SUVmax in predicting PFS in both univariate and multivariate analysis in a cohort of 123 elderly patients with a mix of different treatment regimens. However, neither TLG nor MTV were predictive of OS. Cottereau et al. and Akhtari et al. both assessed the ability of MTV in cohorts of patients consisting of stage I and II disease [12, 21]. Cottereau et al. found that MTV derived from >2.5 SUV was significant in predicting 5-year PFS and OS and was significant in multivariate analysis when assessed with different early disease scoring systems. Akhtari et al. found that MTV and TLG derived from >2.5 SUV thresholding and manual soft tissue contouring were significant predictors of 5-year PFS. Reporting bias is present because a number of studies which did not report any significance did not provide the results required to calculate a HR. The overall HR for MTV in PFS was 2.13 (CI 95% 1.53–2.96) and 2.13 (1.43–3.16) in OS. Both were associated with high levels of heterogeneity, *I*^2^ = 74% for PFS and *I*^2^ = 70% for OS.

**Table 4:** Studies assessing the use of metabolic tumour volume (MTV) and total lesion glycolysis (TLG) and Hodgkin lymphoma (HL)

Author	Year	Patient no.	Stage		Treatment	Events (follow-up cut-off)	Segmentation threshold	MTV/TLG	Cut-off	Predictive univariate analysis HR (95% CI),		Predictive multivariate analysis HR (95% CI), parameters included in multivariate analysis	
			I/II	III/IV						PFS	OS	PFS	OS
Albano [13]^a^	2020	123 Elderly	36	87	ABVD, BEACOPP, R-CHOP, ± RT, RT	Relapse: 51 Died: 37 (no defined cut-off)	41% SUVmax	MTV	89 ml	0.531 (0.294–0.908)	NS	0.555 (0.249–0.965)	NR
								TLG	2199 g	0.544 (0.240–0.963)	NS	0.602 (0.111–0.989)	NR
Lue [34]	2019	42	20	22	Anthracycline-based chemotherapy ± RT	Relapse: 12 Died: 9 (5-year)	41% SUVmax	MTV	183 ml	4.495 (1.434–14.09)	4.500 (1.205–16.81)	NS	NS
Mettler [35]	2019	310		310	eBEACOPP (4 or 6 cycles)	PFS: 16 events OS: 7 events (no defined cut-off)	41% SUVmax, >2.5 SUV, Liver SUVmax, Liver SUVmean	MTV41%	NR	NS	NS	NS	NS
								MTV2.5	NR	NS	NS	NS	NS
								MTVlmax	NR	NS	NS	NS	NS
								MTVlmean	NR	NS	NS	NS	NS
Akhtari*.* [12]	2018	267	267	0	ABVD ± RT	Relapse/refractor = 27 (5-year)	≥2.5 SUV or manually contour	MTV2.5	Continuous	1.00 (1.0007–1.0025)	NR	NR	NR
								TLG2.5	1703 g	1.00 (1.0001–1.0004	NR	NR	NR
								MTVman	NR	1.00 (1.0006–1.0019)	NR	NR	NR
								TLGman	NR	1.00 (1.0001–1.0004)	NR	NR	NR
Cottereau [21]	2018	258	258		ABVD ± RT	PFS: 27 events OS: 12 (5-year)	≥2.5 SUV	MTV	147 ml	5.2 (1.8–14.7)	7.2 (1.6–33.4)	Sig with individual factors, EORTC, GHCS and NCCN	Sig with individual factors, EORTC, GHCS and NCCN
Angelopoulou [14]	2017	162	76	86	ABVD ± BEACOPP, ± RT	PFS: 81% OS: 93% (5-year)	TLG from maximal largest diameter x SUVmax	TLG	<35, 35–100, <100	70 vs. 81 vs. 94%	NR	NR	NR
Kanoun [29]	2015	59			Anthracycline-based 4–6-8 cycles	Relapse: 5 Died: 5 (no defined cut-off)	41% SUVmax	MTV	225 ml	42 vs. 85%	NR	Sig when analysed with tumour change in SUVmax	NR
Song. [43]	2013	127	127		ABVD ± RT	PFS: 85.8% OS: 88.2% (no defined cut-off)	≥2.5 SUV	MTV	198 ml	10.707 (3.098–37.002)	13.201 (2.975–58.579)	13.008 (3.441–49.174) Age, B symptoms, mediastinal bulky disease	15.831 (3.301–75.926 Age, B symptoms, mediastinal bulky disease
Tseng [46]	2012	30	11	19	Standford V, ABVD, VAMP, BEACOPP	Relapse =6 Died: 4 (4-year)	NR	MTV		NS	NS	NR	NR

*NR* not reported; *NS* not significant; *Sig.* significant; *HR* hazard ratio; *CI* confidence interval; *PFS* progressive free survival; *OS* overall survival; *R-CHOP* rituximab cyclophosphamide, doxorubicin hydrochloride, vincristine (Oncovin) and prednisolone; *R-ACVBP* Rituximab, Doxorubicin, Cyclophosphamide, Vindesine, Bleomycin, prednisolone; *RT* radiotherapy; *ABVD* doxorubicin (Adriamycin), bleomycin, vinblastine and dacarbazine; *eBEACOPP* escalated dose bleomycin, etoposide, doxorubicin (Adriamycin), cyclophosphamide, vincristine (Oncovin), procarbazine, and prednisone; *VAMP* vincristine, doxorubicin hydrochloride, methotrexate, prednisolone; *EORTC* European Organisation for Research and Treatment of Cancer; *GHSC* German Hodgkin lymphoma study group; *NCCN* National Comprehensive Cancer Network

^a^The HRs presented as presented in the study but are inverse to the other HRs within the table

Similar to DLBCL, clinical implementation of MTV and TLG in HL depends on reaching a consensus regarding segmentation methodology, each giving different variations in the volumes measured and will be facilitated by an automated process. However, variation in treatment is likely also to play an impact, and this aspect needs assessing in larger multi-centre studies.

## Textural and shape analysis for outcome prediction

Textural analysis or radiomics relates to transformation of images into mineable high-dimensional data permitting invisible feature extraction, analysis and modelling for non-invasive phenotyping and outcome prediction [53]. Radiomic features can be studied in isolation or increasingly are being combined with clinical and genomic features as part of the rapidly expanding field of integrated diagnostics [54].

Aide et al. studied the use of PET/CT-derived textural features, clinical and imaging parameters to predict 2-year PFS in DLBCL patients [10]. They split patients into training (*n* = 105) and validation sets (*n* = 27) and found that Long-Zone High-Grey Level Emphasis (LZHGE) was the only independent predictor when analysed with IPI and MTV. On the validation set, it was found that a high LZHGE > 1,264,925.92 was associated with a 2-year PFS of 60% whereas patients with a low LZGHE had a PFS of 94.1%. The study has some limitations as only the largest area of disease was analysed, a breakdown of disease stage was not presented and 14 patients did not have standard (R-CHOP) therapy. Another study by Aide et al. investigated the diagnostic and prognostic value of axial skeletal textural features derived from PET/CT in patients with DLBCL in a retrospective cohort of 82 patients [11]. The CT dataset was initially contoured using a segmentation threshold of >150 Hounsfield units (HU) with the spinal column and half of the pelvis included. They reported that the first-order parameter skewness had the highest AUC for predicting BMI and that a cut-off value of 1.26 produced a sensitivity, specificity, PPV and NPV of 82, 82, 62 and 93%, respectively. In addition, a skewness value of <1.26 was associated with a greater 2-year PFS and OS. This was true even for 60 patients without BMI. The study had a low event rate (22 patients had BMI), which limits the ability to create a robust prognostic model.

Lue et al. investigated the use of 11 first-order, 39 higher-order features and 400 wavelet features for predicting PFS and OS in 42 HL patients (20 stage I/II, 22 stage III/IV) with 21 events within the cohort (12 relapses, 9 deaths) [34]. They found 173 radiomic features, which were significant predictors of progression after correction for multiple testing. To avoid multicollinearity, they only selected the top two features according to the AUC from each group to be included in the univariate and multivariate analysis. MTV was selected based on previous studies. They demonstrated that SUV kurtosis, stage and intensity non-uniformity (INU) derived from Grey-Level Run Length Matrix (GLRLM) were independent predictors of PFS and only disease stage and INU derived from GLRLM were independent predictors of OS.

Decazes et al. retrospectively studied PET/CT scans of 215 DLBCL patients to assess the utility of total tumour surface (TTS) and tumour volume surface ratio (TVSR) as predictive biomarkers [23], TVSR being the ratio between MTV and TTS. MTV had the highest AUC for both OS and PFS (0.71 and 0.67) when compared to TTS (0.69 and 0.66) and TVSR (0.65 and 0.61) [23]. It was reported that TVSR, MTV, IPI and type of chemotherapy were all independent prognostic parameters. Milogrom et al. investigated the use of a support vector machine model based on first and second-order radiomic features derived from baseline PET/CT to predict relapse or refractory disease in 167 stage I-II HL patients with mediastinal involvement [37]. Ten of the groups formed the training set, and two were designated the validation set with each group containing a single event (*n* = 12). Five features were selected as the most predictive (SUVmax, MTV, InformationMeasureCorr1, InformationMeasureCorr2 and InverseVariance derived from GLCM 2.5). InformationMeasureCorr1 and InformationMeasureCorr2 are the first and second measures of theoretic correlation and Inverse-Variance is weighting of random variables to minimise variance. By combining these features, the AUC for predicting relapse for patients with mediastinal disease was 0.95. This outperformed TLG and MTV. This work highlights the potential for using AI-methods in lymphoma assessment. However, the study is limited to HL with mediastinal involvement with again small numbers of events.

Senjo et al. demonstrated that a high metabolic heterogeneity (MH) was a predictor of 5-year PFS and OS in DLBCL across both training (*n* = 86) and validation cohorts (*n* = 64) treated at two centres [41]. They found that MH remained a significant predictor for 5-year OS for both cohorts when analysed in multivariate analysis with an ECOG score of >2, and an LDH with a relative risk of 4.75 (95% CI 1.25–18.1) and relative risk of 4.92 (95% CI 1.09–17.03) in the training and validation groups, respectively. A model was created which combined MH and MTV, which successfully risk stratified the combined training and validation cohorts into three risk groups: low MH and low MTV, low MH and high MTV or high MH and low MTV, and high MH and high MTV, with the 5-year OS being 90.4 vs. 69.5 vs. 34.8%, respectively; *P* < 0.001 and 5-year PFS, 84.1 vs. 43.6 vs. 27.0%, *P* < 0.001 respectively.

## Current limitations and future challenges

**Fig. 8:**
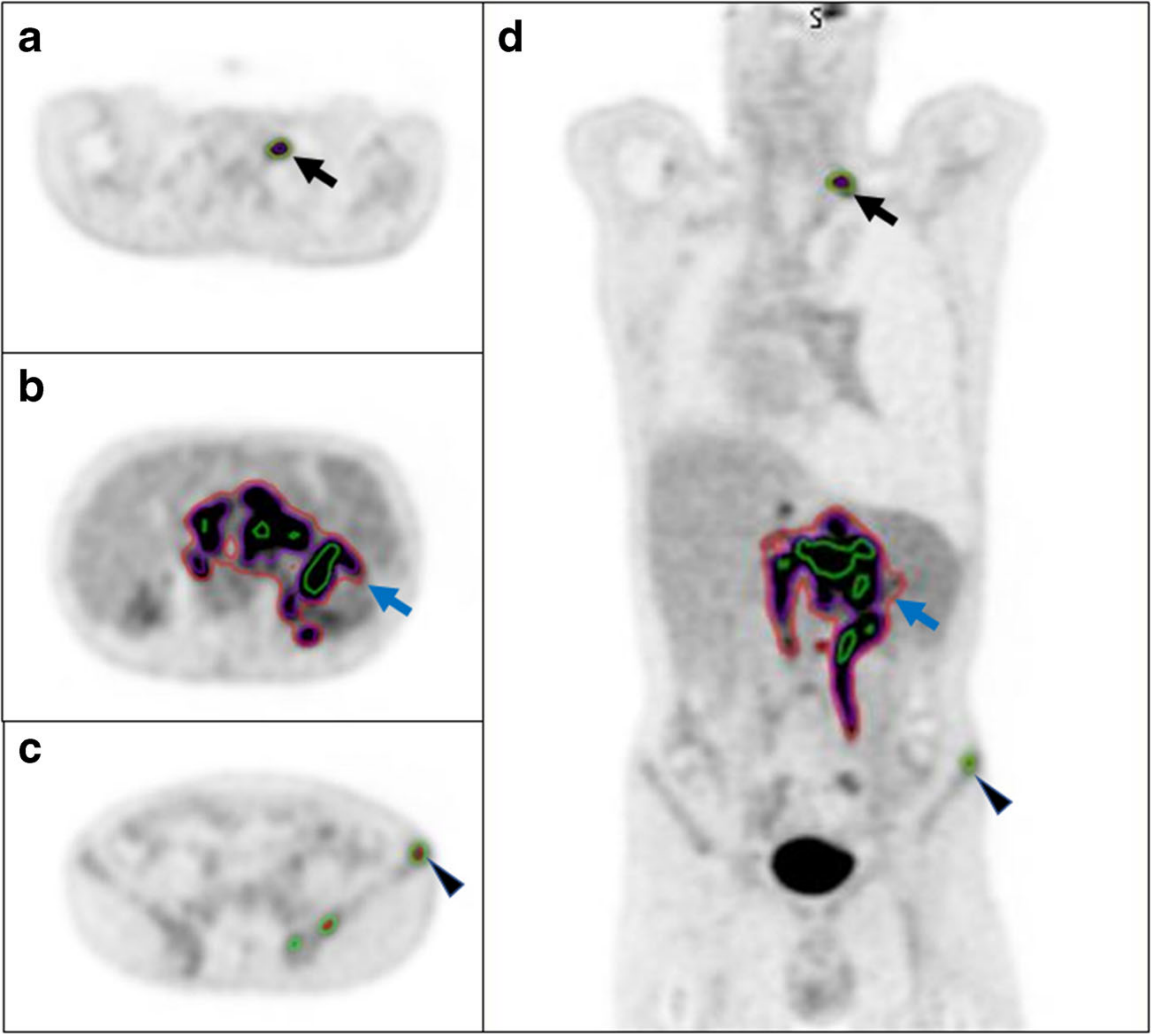
Select axial (**a**–**c**) and coronal slices (**d**) from an FDG PET/CT study from a patient with DLBCL demonstrating three different contouring methods (green = 41% SUVmax; red = 1.5 x SUVmean of the liver; purple = 4.0 SUV). For smaller lesions, the 41% SUVmax contour is larger than the other two methods, black arrow and arrowhead. For larger more heterogenous lesions, the 41% SUVmax is the smallest of the three contours (blue arrow)

One issue needing to be addressed when using imaging parameters derived from PET for predictive modelling is the relatively low spatial resolution, which influences how much of the avidity is included within a volume when different thresholding techniques are utilised (Fig. 8) [55]. Meignan et al. used a phantom model to validate their MTV thresholding method for a patient cohort [56]. They found that a 41% SUVmax threshold gave the best concordance between contoured and actual volumes. 41% SUVmax thresholding also gave the best agreement between reviewers using the Lin concordance correlation coefficient (pc) (ρc = 0.986, CI 0.97–0.99). However, for successful clinical implementation, the time it takes to implement as well as the accuracy of the thresholding method needs be considered. The use of a semi-automated method such as the one reported by Burggraaff et al. [57] or a deep learning derived volume as reported by Capobianco et al. is required [15]. Predictive models also need to be tested and adapted for new treatments or histological markers [58]. The ability to be able to predict worse outcomes could allow for future treatment stratification. There is an area of unmet need with few active studies at present. There are currently only two open/recruiting studies listed on clinicaltrials.gov assessing PET/CT parameters for outcome prediction in DLBCL, and no registered studies assessing outcomes in HL patients.

Other important limitations of the published work highlighted in this systematic review are variability in methodology and lack of external validation (Table 5). This presents a number of opportunities for the future (Table 5). Further study into the use of AI for imaging-based outcome prediction in lymphoma which may permit more accurate prediction of prognosis/treatment outcome is needed. This might also facilitate more efficient image analysis and actionable clinical decision support potentially guiding tailored treatment for individual patients. However, there is the requirement for large volumes of data necessary to train algorithms which can then be vigorously validated for reproducibility and generalizability which will require cross-institutional collaboration via imaging networks to support the establishment of multi-centre trials. Implementation studies and health economic research will also be critical for clinical adoption by demonstrating that any AI application is reliable and value-based.

**Table 5:** Limitations of the current literature and opportunities for future work

Limitation	Opportunity
1. Relatively small retrospective cohorts with limited events	Establishing multi-centre networks for future larger-scale studies
2. Multiple segmentation techniques used	Consensus on segmentation technique for MTV and TLG and development of automated AI-methods which are implemented within reporting software by manufacturers
3. Single site models using a single dataset	Internal and external validation should be routinely performed and facilitated by networks
4. Varying predictive end points	Consensus on clinically relevant predictors
5. Small numbers of papers using non-linear analysis	Using different machine learning and deep learning models to aid in imaging analysis and outcome prediction

All the described limitations have led to a medium and high risk of bias within the literature as evaluated with our QUIPS tool. The decision to retain papers with a high risk of bias was taken as it was felt that this itself would introduce bias into the review. However, this does mean the results need to be interpretated with caution. Further work in this area is clearly warranted and efforts should be made when designing future studies to carefully consider the methodology employed so as to minimise the risk of bias which is prevalent in this field of work to date.

## Conclusion

Multiple reports suggest the potential utility of various PET/CT-derived imaging parameters in lymphoma outcome modelling. Most studies are retrospective and lack external validation of described models. Robustness across different scanning protocols and institutions has also not been verified, and clinical implementation remains a future aspiration. AI techniques may offer a potential solution to some limitations of predictive modelling in this clinical scenario and warrant further evaluation.

## Supplementary Information

ESM 1 (XLSX 13 kb)

ESM 2 (DOCX 55 kb)

## References

[1] Shanbhag S, Ambinder RF (2018). Hodgkin lymphoma: a review and update on recent progress. CA Cancer J Clin.

[2] SEER. SEER Cancer Statistics Review. 1975–2016. 2018.

[3] Armitage JO, Gascoyne RD, Lunning MA, Cavalli F (2017). Non-Hodgkin lymphoma. Lancet..

[4] Horvat M, Zadnik V, Šetina TJ, Boltežar L, Goličnik JP, Novaković S (2018). Diffuse large B-cell lymphoma: 10 years’ real-world clinical experience with rituximab plus cyclophosphamide, doxorubicin, vincristine and prednisolone. Oncol Lett.

[5] El-Galaly TC, Gormsen LC, Hutchings M (2018). PET/CT for staging; past, present, and future. Semin Nucl Med.

[6] Liberati A, Altman DG, Tetzlaff J, Mulrow C, Gøtzsche PC, Ioannidis JPA (2009). The PRISMA statement for reporting systematic reviews and meta-analyses of studies that evaluate health care interventions: explanation and elaboration. J Clin Epidemiol.

[7] Hayden JA, van der Windt DA, Cartwright JL, Co P (2013). Research and reporting methods annals of internal medicine assessing bias in studies of prognostic factors. Ann Intern Med.

[8] Grooten WJA, Tseli E, Äng BO, Boersma K, Stålnacke B-M, Gerdle B (2019). Elaborating on the assessment of the risk of bias in prognostic studies in pain rehabilitation using QUIPS—aspects of interrater agreement. Diagn Progn Res.

[9] Adams HJA, de Klerk JMH, Fijnheer R, Heggelman BGF, Dubois SV, Nievelstein RAJ (2015). Prognostic superiority of the National Comprehensive Cancer Network International Prognostic Index over pretreatment whole-body volumetric-metabolic FDG-PET/CT metrics in diffuse large B-cell lymphoma. Eur J Haematol.

[10] Aide N, Fruchart C, Nganoa C, Gac A, Lasnon C (2020). Baseline 18 F-FDG PET radiomic features as predictors of 2-year event-free survival in diffuse large B cell lymphomas treated with immunochemotherapy. Eur Radiol.

[11] Aide N, Talbot M, Fruchart C, Damaj G, Lasnon C (2018). Diagnostic and prognostic value of baseline FDG PET/CT skeletal textural features in diffuse large B cell lymphoma. Eur J Nucl Med Mol Imaging.

[12] Akhtari M, Milgrom SA, Pinnix CC, Reddy JP, Dong W, Smith GL (2018). Reclassifying patients with early-stage Hodgkin lymphoma based on functional radiographic markers at presentation. Blood..

[13] Albano D, Mazzoletti A, Spallino M, Muzi C, Zilioli VR, Pagani C (2020). Prognostic role of baseline 18F-FDG PET/CT metabolic parameters in elderly HL: a two-center experience in 123 patients. Ann Hematol.

[14] Angelopoulou MK, Mosa E, Pangalis GA, Rondogianni P, Chatziioannou S, Prassopoulos V (2017). The significance of PET/CT in the initial staging of Hodgkin lymphoma: experience outside clinical trials. Anticancer Res.

[15] Capobianco N, Meignan MA, Cottereau A-S, Vercellino L, Sibille L, Spottiswoode B, et al. Deep learning FDG uptake classification enables total metabolic tumor volume estimation in diffuse large B-cell lymphoma. J Nucl Med. 2020;62:30–36. 10.2967/jnumed.120.242412 PMC8679589 32532925

[16] Ceriani L, Gritti G, Cascione L, Pirosa MC, Polino A, Ruberto T (2020). SAKK38/07 study: integration of baseline metabolic heterogeneity and metabolic tumor volume in DLBCL prognostic model. Blood Adv.

[17] Chang C-C, Cho S-F, Chuang Y-W, Lin C-Y, Chang S-M, Hsu W-L (2017). Prognostic significance of total metabolic tumor volume on 18F-fluorodeoxyglucose positron emission tomography/ computed tomography in patients with diffuse large B-cell lymphoma receiving rituximab-containing chemotherapy. Oncotarget..

[18] Chang C, Cho S, Tu H, Lin C, Chuang Y, Chang S (2017). Tumor and bone marrow uptakes on [18F] fluorodeoxyglucose positron emission tomography/computed tomography predict prognosis in patients with diffuse large B-cell lymphoma receiving rituximab-containing chemotherapy. Med..

[19] Chihara D, Oki Y, Onoda H, Taji H, Yamamoto K, Tamaki T (2011). High maximum standard uptake value (SUVmax) on PET scan is associated with shorter survival in patients with diffuse large B cell lymphoma. Int J Hematol.

[20] Cottereau AS, Nioche C, Dirand AS, Clerc J, Morschhauser F, Casasnovas O (2020). 18F-FDG PET dissemination features in diffuse large B-cell lymphoma are predictive of outcome. J Nucl Med.

[21] Cottereau AS, Versari A, Loft A, Casasnovas O, Bellei M, Ricci R (2018). Prognostic value of baseline metabolic tumor volume in early-stage Hodgkin lymphoma in the standard arm of the H10 trial. Blood..

[22] Cottereau AS, Lanic H, Mareschal S, Meignan M, Vera P, Tilly H (2016). Molecular profile and FDG-PET/CT Total metabolic tumor volume improve risk classification at diagnosis for patients with diffuse large B-cell lymphoma. Clin Cancer Res.

[23] Decazes P, Becker S, Toledano MN, Vera P, Desbordes P, Jardin F (2018). Tumor fragmentation estimated by volume surface ratio of tumors measured on 18F-FDG PET/CT is an independent prognostic factor of diffuse large B-cell lymphoma. Eur J Nucl Med Mol Imaging.

[24] Esfahani SA, Heidari P, Halpern EF, Hochberg EP, Palmer EL, Mahmood U (2013). Baseline total lesion glycolysis measured with (18)F-FDG PET/CT as a predictor of progression-free survival in diffuse large B-cell lymphoma: a pilot study. Am J Nucl Med Mol Imaging.

[25] Gallicchio R, Mansueto G, Simeon V, Nardelli A, Guariglia R, Capacchione D (2014). F-18 FDG PET/CT quantization parameters as predictors of outcome in patients with diffuse large B-cell lymphoma. Eur J Haematol.

[26] Huang H, Xiao F, Han X, Zhong L, Zhong H, Xu L (2016). Correlation of pretreatm ent 18F-FDG uptake with clinicopathological factors and prognosis in patients with newly diagnosed diffuse large B-cell lymphoma. Nucl Med Commun.

[27] Ilyas H, Mikhaeel NG, Dunn JT, Rahman F, Möller H, Smith D (2019). Is there an optimal method for measuring baseline metabolic tumor volume in diffuse large B cell lymphoma?. Eur J Nucl Med Mol Imaging.

[28] Jegadeesh N, Rajpara R, Esiashvili N, Shi Z, Liu Y, Okwan-Duodu D (2015). Predictors of local recurrence after rituximab-based chemotherapy alone in stage III and IV diffuse large b-cell lymphoma: guiding decisions for consolidative radiation. Int J Radiat Oncol Biol Phys.

[29] Kanoun S, Tal I, Berriolo-Riedinger A, Rossi C, Riedinger JM, Vrigneaud JM (2015). Influence of software tool and methodological aspects of total metabolic tumor volume calculation on baseline [18F] FDG PET to predict survival in Hodgkin lymphoma. PLoS One.

[30] Kim CY, Hong CM, Kim DH, Son SH, Jeong SY, Lee SW (2013). Prognostic value of whole-body metabolic tumour volume and total lesion glycolysis measured on 18F-FDG PET/CT in patients with extranodal NK/T-cell lymphoma. Eur J Nucl Med Mol Imaging.

[31] Kim TM, Paeng JC, Chun IK, Keam B, Jeon YK, Lee SH (2013). Total lesion glycolysis in positron emission tomography is a better predictor of outcome than the International Prognostic Index for patients with diffuse large B cell lymphoma. Cancer..

[32] Kwon SH, Kang DR, Kim J, Yoon JK, Lee SJ, Jeong SH (2016). Prognostic value of negative interim 2-[ 18 F]-fluoro-2-deoxy-d-glucose PET/CT in diffuse large B-cell lymphoma. Clin Radiol.

[33] Lanic H, Mareschal S, Mechken F, Picquenot JM, Cornic M, Maingonnat C (2012). Interim positron emission tomography scan associated with international prognostic index and germinal center B cell-like signature as prognostic index in diffuse large B-cell lymphoma. Leuk Lymphoma.

[34] Lue KH, Wu YF, Liu SH, Hsieh TC, Chuang KS, Lin HH (2019). Prognostic value of pretreatment radiomic features of 18F-FDG PET in patients with Hodgkin lymphoma. Clin Nucl Med.

[35] Mettler J, Müller H, Voltin CA, Baues C, Klaeser B, Moccia A (2019). Metabolic tumor volume for response prediction in advanced-stage hodgkin lymphoma. J Nucl Med.

[36] Mikhaeel NG, Smith D, Dunn JT, Phillips M, Møller H, Fields PA (2016). Combination of baseline metabolic tumour volume and early response on PET/CT improves progression-free survival prediction in DLBCL. Eur J Nucl Med Mol Imaging.

[37] Milgrom SA, Elhalawani H, Lee J, Wang Q, Mohamed ASR, Dabaja BS (2019). A PET radiomics model to predict refractory mediastinal Hodgkin lymphoma. Sci Rep.

[38] Miyazaki Y, Nawa Y, Miyagawa M, Kohashi S, Nakase K, Yasukawa M (2013). Maximum standard uptake value of 18F-fluorodeoxyglucose positron emission tomography is a prognostic factor for progression-free survival of newly diagnosed patients with diffuse large B cell lymphoma. Ann Hematol.

[39] Park S, Moon SH, Park LC, Hwang DW, Ji JH, Maeng CH (2012). The impact of baseline and interim PET/CT parameters on clinical outcome in patients with diffuse large B cell lymphoma. Am J Hematol.

[40] Sasanelli M, Meignan M, Haioun C, Berriolo-Riedinger A, Casasnovas RO, Biggi A (2014). Pretherapy metabolic tumour volume is an independent predictor of outcome in patients with diffuse large B-cell lymphoma. Eur J Nucl Med Mol Imaging.

[41] Senjo H, Hirata K, Izumiyama K, Minauchi K, Tsukamoto E, Itoh K (2020). High metabolic heterogeneity on baseline 18FDG-PET/CT scan as a poor prognostic factor for newly diagnosed diffuse large B-cell lymphoma. Blood Adv.

[42] Song MK, Yang DH, Lee GW, Lim SN, Shin S, Pak KJ (2016). High total metabolic tumor volume in PET/CT predicts worse prognosis in diffuse large B cell lymphoma patients with bone marrow involvement in rituximab era. Leuk Res.

[43] Song MK, Chung JS, Lee JJ, Jeong SY, Lee SM, Hong JS (2013). Metabolic tumor volume by positron emission tomography/computed tomography as a clinical parameter to determine therapeutic modality for early stage Hodgkin’s lymphoma. Cancer Sci.

[44] Song MK, Chung JS, Shin HJ, Lee SM, Lee SE, Lee HS (2012). Clinical significance of metabolic tumor volume by PET/CT in stages II and III of diffuse large B cell lymphoma without extranodal site involvement. Ann Hematol.

[45] Toledano MN, Desbordes P, Banjar A, Gardin I, Vera P, Ruminy P (2018). Combination of baseline FDG PET/CT total metabolic tumour volume and gene expression profile have a robust predictive value in patients with diffuse large B-cell lymphoma. Eur J Nucl Med Mol Imaging.

[46] Tseng D, Rachakonda LP, Su Z, Advani R, Horning S, Hoppe RT, et al. Interim-treatment quantitative PET parameters predict progression and death among patients with hodgkin’s disease. Radiat Oncol. 2012;7:5. 10.1186/1748-717X-7-5 PMC3398283 22260710

[47] Xie M, Zhai W, Cheng S, Zhang H, Xie Y, He W (2016). Predictive value of F-18 FDG PET/CT quantization parameters for progression-free survival in patients with diffuse large B-cell lymphoma. Hematology..

[48] Zhang YY, Song L, Zhao MX, Hu K (2019). A better prediction of progression-free survival in diffuse large B-cell lymphoma by a prognostic model consisting of baseline TLG and %ΔSUVmax. Cancer Med.

[49] Zhou M, Chen Y, Huang H, Zhou X, Liu J, Huang G (2016). Prognostic value of total lesion glycolysis of baseline F-fluorodeoxyglucose positron emission tomography/ computed tomography in diffuse large B-cell lymphoma other factors including MTV, National Comprehensive Cancer Network International Prognostic Ind. Oncotarget..

[50] Fletcher JW, Kinahan PE (2010). PET/CT standardized uptake values (SUVs) in clinical practice and assessing response to therapy. Semin Ultrasound CT MR.

[51] Adams MC, Turkington TG, Wilson JM, Wong TZ (2010). A systematic review of the factors affecting accuracy of SUV measurements. Am J Roentgenol.

[52] Fletcher JW, Kinahan PE. PET/CT standardized uptake values (SUVs) in clinical practice and assessing response to therapy. Semin Ultrasound CT MR. 2010;31:496–505. 10.1053/j.sult.2010.10.001 PMC3026294 21147377

[53] Lambin P, Leijenaar RTH, Deist TM, Peerlings J, De Jong EEC, Van Timmeren J (2017). Radiomics: the bridge between medical imaging and personalized medicine. Nat Rev Clin Oncol.

[54] Lundström CF, Gilmore HL, Ros PR (2017). Integrated diagnostics: the computational revolution catalyzing cross-disciplinary practices in radiology, pathology, and genomics. Radiology..

[55] Barrington SF, Meignan M (2019). Time to prepare for risk adaptation in lymphoma by standardizing measurement of metabolic tumor burden. J Nucl Med.

[56] Meignan M, Sasanelli M, Casasnovas RO, Luminari S, Fioroni F, Coriani C (2014). Metabolic tumour volumes measured at staging in lymphoma: methodological evaluation on phantom experiments and patients. Eur J Nucl Med Mol Imaging.

[57] Burggraaff CN, Rahman F, Kaßner I, Pieplenbosch S, Barrington SF, Jauw YWS (2020). Optimizing workflows for fast and reliable metabolic tumor volume measurements in diffuse large B cell lymphoma. Mol Imaging Biol.

[58] Vercellino L, Cottereau A-S, Casasnovas O, Tilly H, Feugier P, Chartier L (2020). High total metabolic tumor volume at baseline predicts survival independent of response to therapy. Blood..

