# Supplementary material for: Baseline PET/CT imaging parameters for prediction of treatment outcome in Hodgkin and diffuse large B cell lymphoma: a systematic review
Source: Eur J Nucl Med Mol Imaging. 2021 Feb 18;48(10):3198–220. doi: 10.1007/s00259-021-05233-2 (PMC8426243; doi:10.1007/s00259-021-05233-2)
Supplement: Supplementary file 2 — (DOCX 55 kb) [file 259_2021_5233_MOESM2_ESM.docx]

| **Study** | **Participation** | **Attrition** | **Prognostic Measurement** | **Outcome Measurement** | **Confounding** | **Analysis and Reporting** |
| --- | --- | --- | --- | --- | --- | --- |
| **Adams** [9] | Moderate | Moderate | Moderate | Moderate | Moderate | Low |
| **Aide** [10] | Low | Moderate | High | High | Moderate | Moderate |
| **Aide** [11] | Moderate | Moderate | Moderate | Moderate | Moderate | Low |
| **Akhtari** [12] | Moderate | Moderate | Moderate | Moderate | Moderate | Low |
| **Albano** [13] | Moderate | Moderate | Moderate | Moderate | Moderate | Low |
| **Angelopulou** [14] | High | Moderate | Moderate | Moderate | High | High |
| **Capobianco** [15] | Moderate | Moderate | High | High | High | High |
| **Ceriani** [16] | Moderate | Moderate | High | High | Moderate | Low |
| **Chang** [17] | Moderate | Moderate | Moderate | Moderate | Moderate | Low |
| **Chang** [18] | Moderate | Moderate | Moderate | Moderate | Moderate | Low |
| **Chihara** [19] | Moderate | Moderate | Moderate | Moderate | Moderate | Low |
| **Cottereau** [20] | Moderate | Moderate | High | High | Moderate | Low |
| **Cottereau** [21] | Moderate | Moderate | Moderate | Moderate | Moderate | Low |
| **Cottereau** [22] | Moderate | Moderate | Moderate | Moderate | Moderate | Low |
| **Decazes** [23] | Low | Moderate | Moderate | Moderate | Moderate | Low |
| **Esfahani** [24] | Moderate | Moderate | High | High | High | High |
| **Gallicchio** [25] | High | High | High | High | High | High |
| **Huang** [26] | Moderate | Moderate | Moderate | Moderate | Moderate | Low |
| **Ilyas** [27] | High | Moderate | Moderate | Moderate | High | High |
| **Jegadesh** [28] | Moderate | Moderate | Moderate | Moderate | Moderate | Low |
| **Kanoun** [29] | Moderate | Moderate | High | High | Moderate | Low |
| **Kim** [30] | Moderate | Moderate | Moderate | Moderate | Moderate | Low |
| **Kim** [31] | Moderate | Moderate | Moderate | Moderate | Moderate | Low |
| **Kwon** [32] | High | Moderate | Moderate | Moderate | Moderate | Low |
| **Lanic** [33] | High | Moderate | Moderate | Moderate | High | High |
| **Lue** [34] | Moderate | Moderate | Moderate | Moderate | Moderate | Low |
| **Mettler** [35] | Moderate | High | Moderate | Moderate | Moderate | Low |
| **Mikhaeel** [36] | Moderate | Moderate | Moderate | Moderate | Moderate | Low |
| **Milgrom** [37] | Moderate | High | Moderate | Moderate | High | Low |
| **Miyazaki** [38] | Moderate | Moderate | Moderate | Moderate | Moderate | Low |
| **Park** [39] | Moderate | High | Moderate | Moderate | High | High |
| **Sasanelli** [40] | Moderate | Moderate | Moderate | Moderate | Moderate | Low |
| **Senjo** [41] | Moderate | Moderate | Moderate | Moderate | High | Low |
| **Song** [42] | Moderate | Moderate | Moderate | Moderate | Moderate | Low |
| **Song** [43] | Moderate | Moderate | Moderate | Moderate | Moderate | Low |
| **Song** [44] | Moderate | Moderate | Moderate | Moderate | Moderate | Low |
| **Toledano** [45] | Moderate | Moderate | Moderate | Moderate | Moderate | Low |
| **Tseng** [46] | Moderate | Moderate | High | High | High | Low |
| **Xie** [47] | High | High | Moderate | Moderate | Moderate | Low |
| **Zhang** [48] | Moderate | Moderate | Moderate | Moderate | Moderate | Low |
| **Zhou** [49] | Moderate | Moderate | Moderate | Moderate | Moderate | Low |

**Supplemental Table 2**: Break down of consensus risk of bias gradings across 6 domains using the Quality in Prognosis Studies (QUIPS) tool for all studies included within the systematic review.
